# Supplementary material for: Discovery of Novel Thiophene/Hydrazones: In Vitro and In Silico Studies against Pancreatic Cancer
Source: Pharmaceutics. 2023 May 9;15(5):1441. doi: 10.3390/pharmaceutics15051441 (PMC10223062; doi:10.3390/pharmaceutics15051441)
Supplement: Supplementary file 1 [file pharmaceutics-15-01441-s001.zip › pharmaceutics-2328857-supplementary.pdf]

SUPPLEMENTARY MATERIAL

Discovery of Novel Thiophene/Hydrazones: *In vitro* and *In silico* Studies agains pancreatic cancer

Goknil Pelin Coskun[a], Rengin Reis[b], Vladimir Dobričić[c], Jelena Bošković[c], Yagmur Ozhan[d], Hande Sipahi[d], Zafer Sahin[e], Seref Demirayak[f]

[a]*Acibadem Mehmet Ali Aydinlar University, Faculty of Pharmacy, Department of Pharmaceutical Chemistry, Istanbul, Turkey*

[b]*Acibadem Mehmet Ali Aydinlar University, Faculty of Pharmacy, Department of Pharmaceutical Toxicology, Istanbul, Turkey*

[c] *University of Belgrade-Faculty of Pharmacy, Department of Pharmaceutical Chemistry, Belgrade, Serbia*

[d] *Yeditepe University, Faculty of Pharmacy, Department of Pharmaceutical Toxicology, Istanbul, Turkey*

[e]*University of Health Sciences, Hamidiye Faculty of Pharmacy, Department of Pharmaceutical Chemistry, Istanbul, Turkey*

[f]*Kocaeli Health and Technology University, Faculty of Pharmacy, Department of Pharmaceutical Chemistry, Kocaeli, Turkey*

*Corresponding author: GP Coskun (pelin.coskun@acibadem.edu.tr)

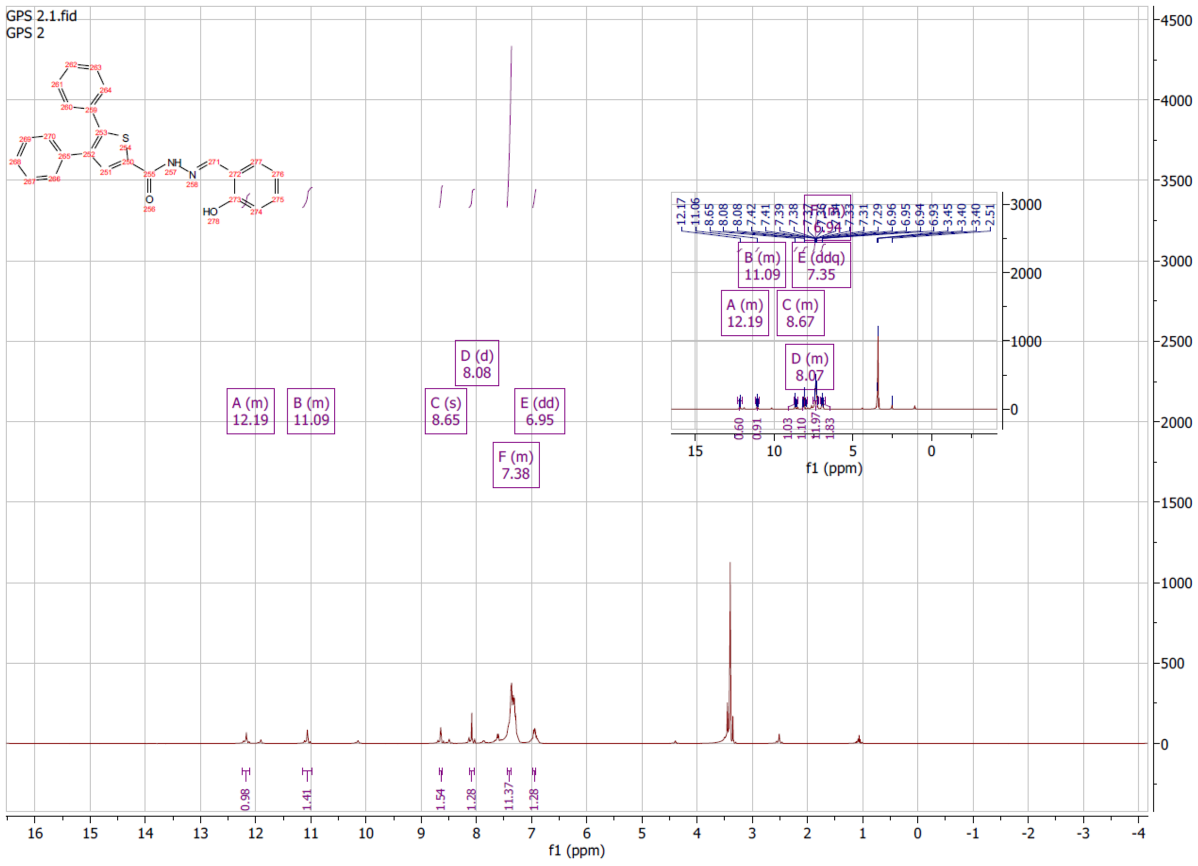

Figure S.1.-^{1}H-NMR spectrum of compound 7a

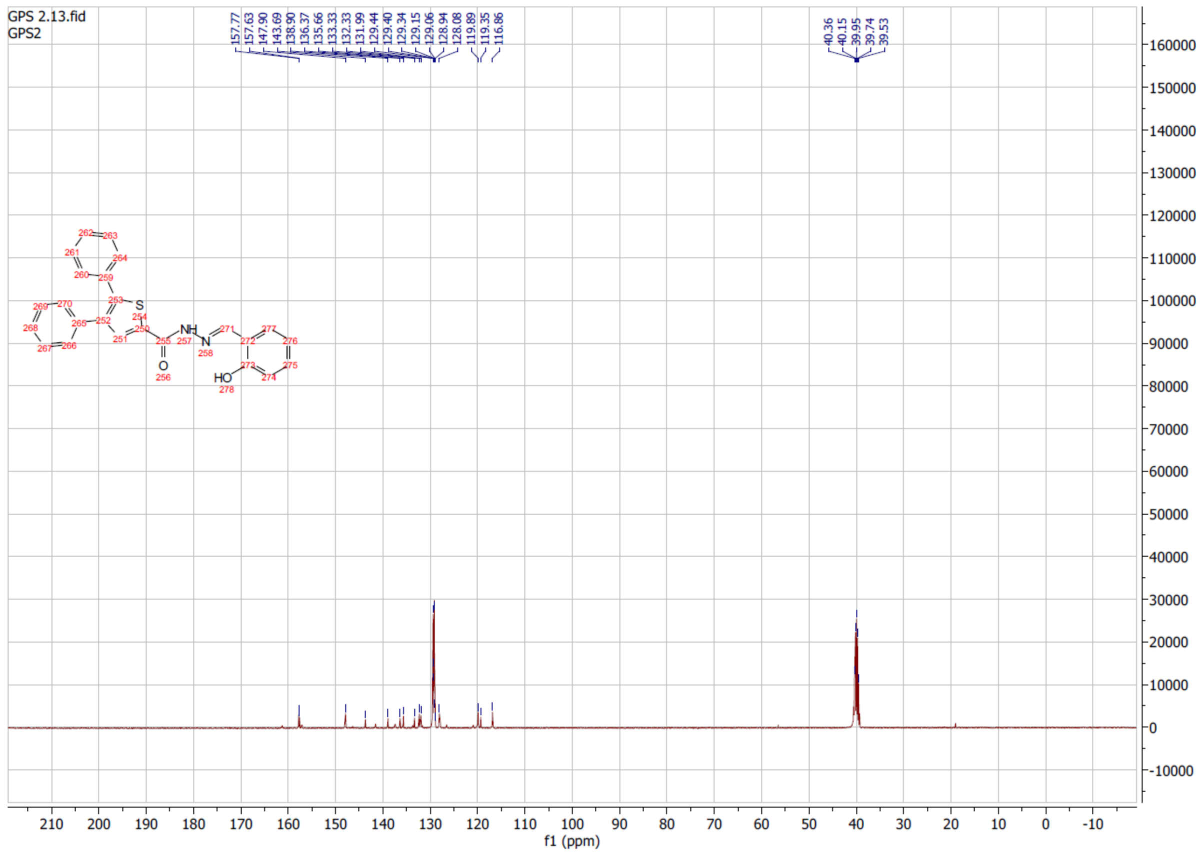

Figure S.2. ^{13}C-NMR spectrum of compound 7a

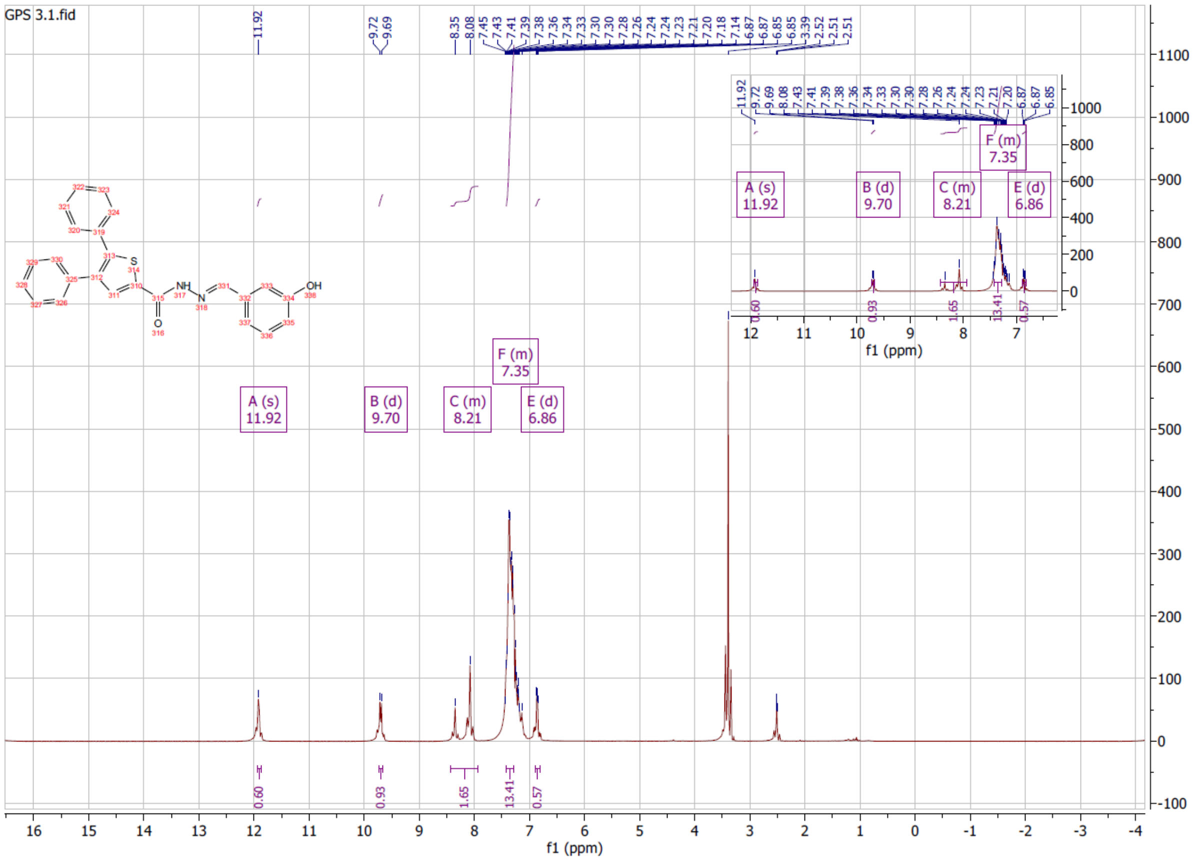

Figure S.3.-^{1}H-NMR spectrum of compound 7b

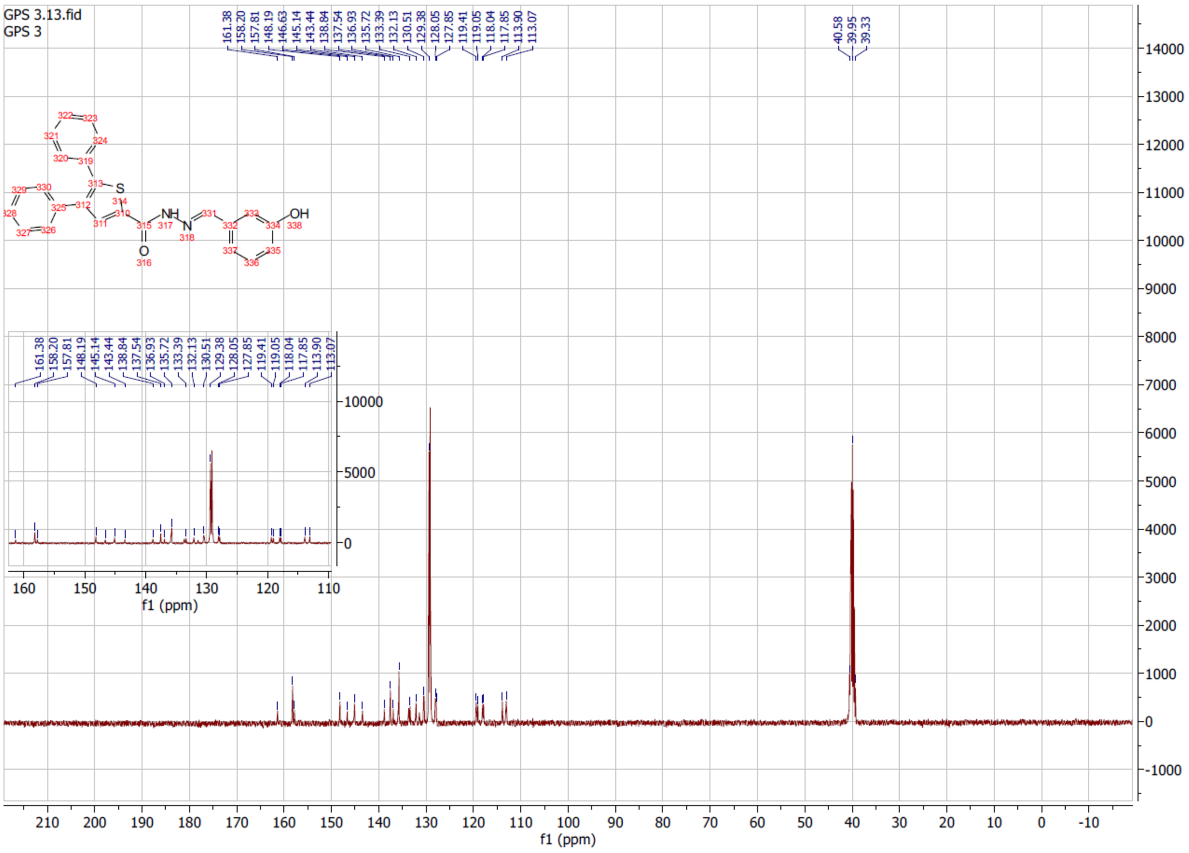

Figure S.4. ^{13}C-NMR spectrum of compound 7b

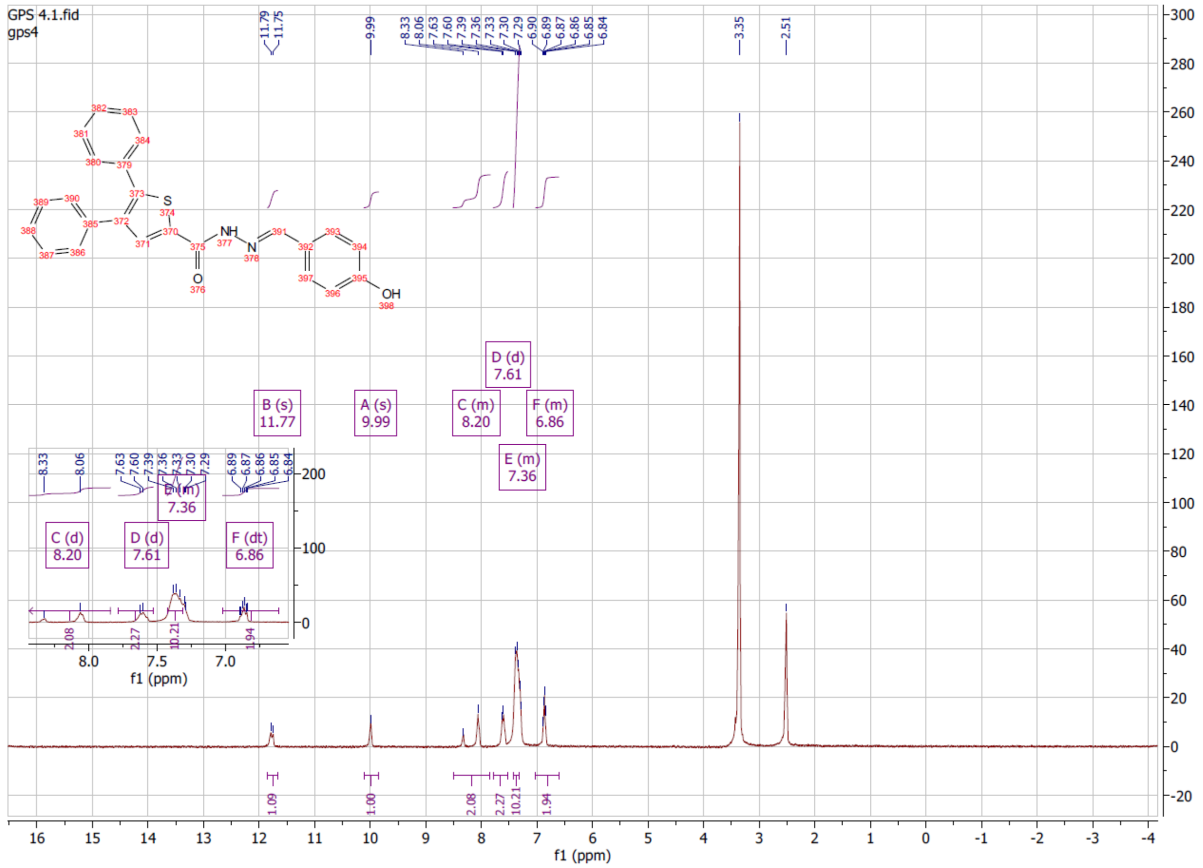

Figure S.5.-^{1}H-NMR spectrum of compound 7c

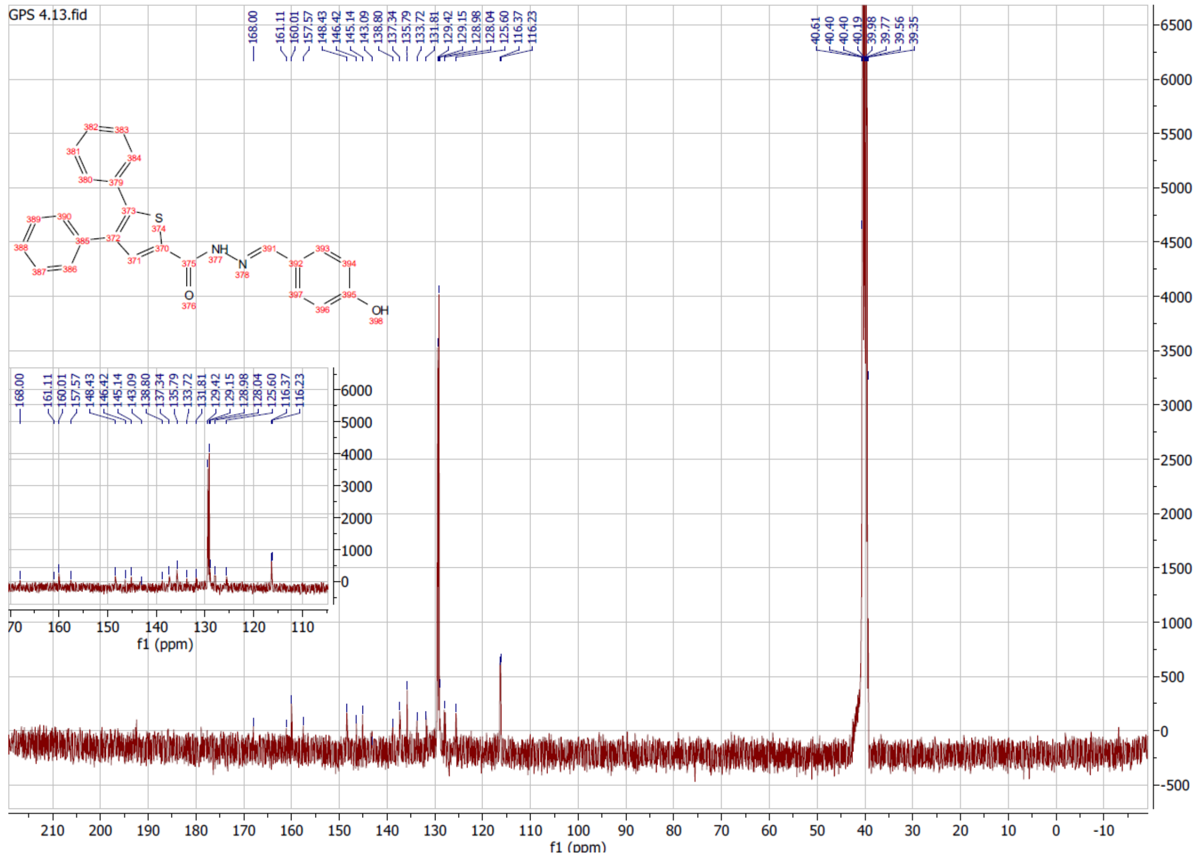

Figure S.6. ^{13}C-NMR spectrum of compound 7c

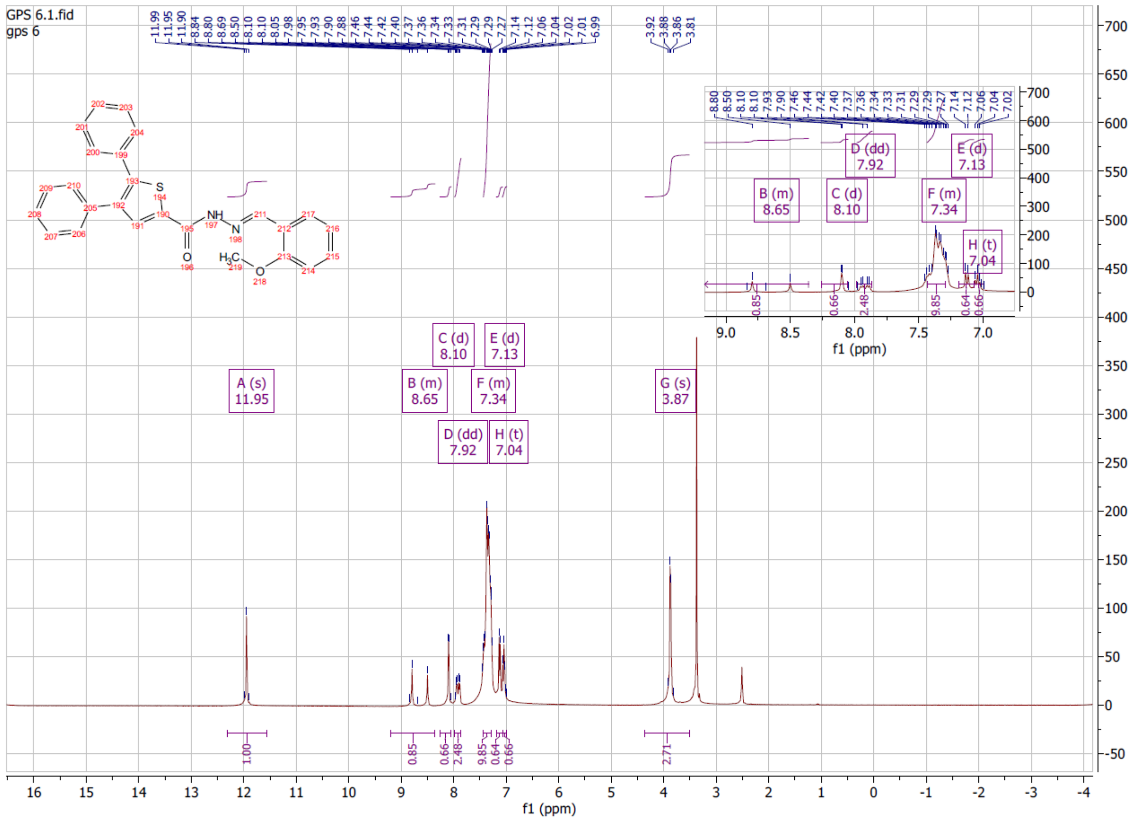

Figure S.7.-^{1}H-NMR spectrum of compound 7d

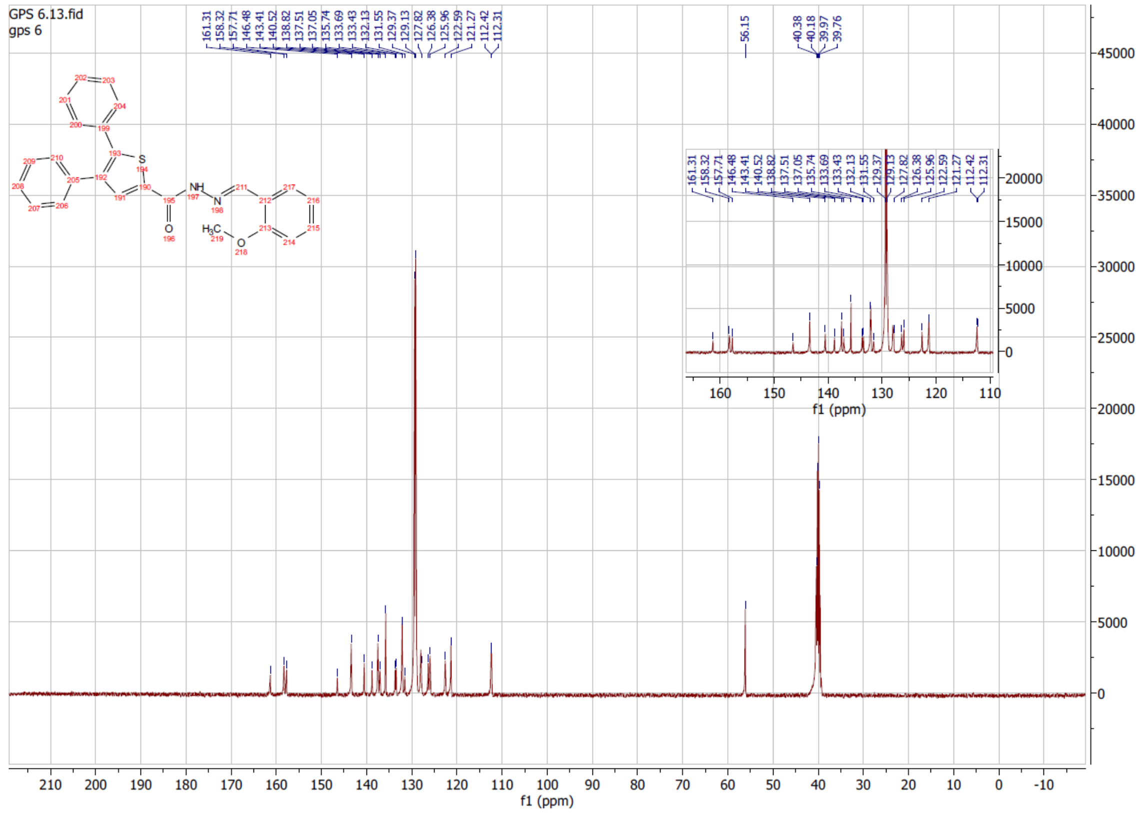

Figure S.8. ^{13}C-NMR spectrum of compound 7d

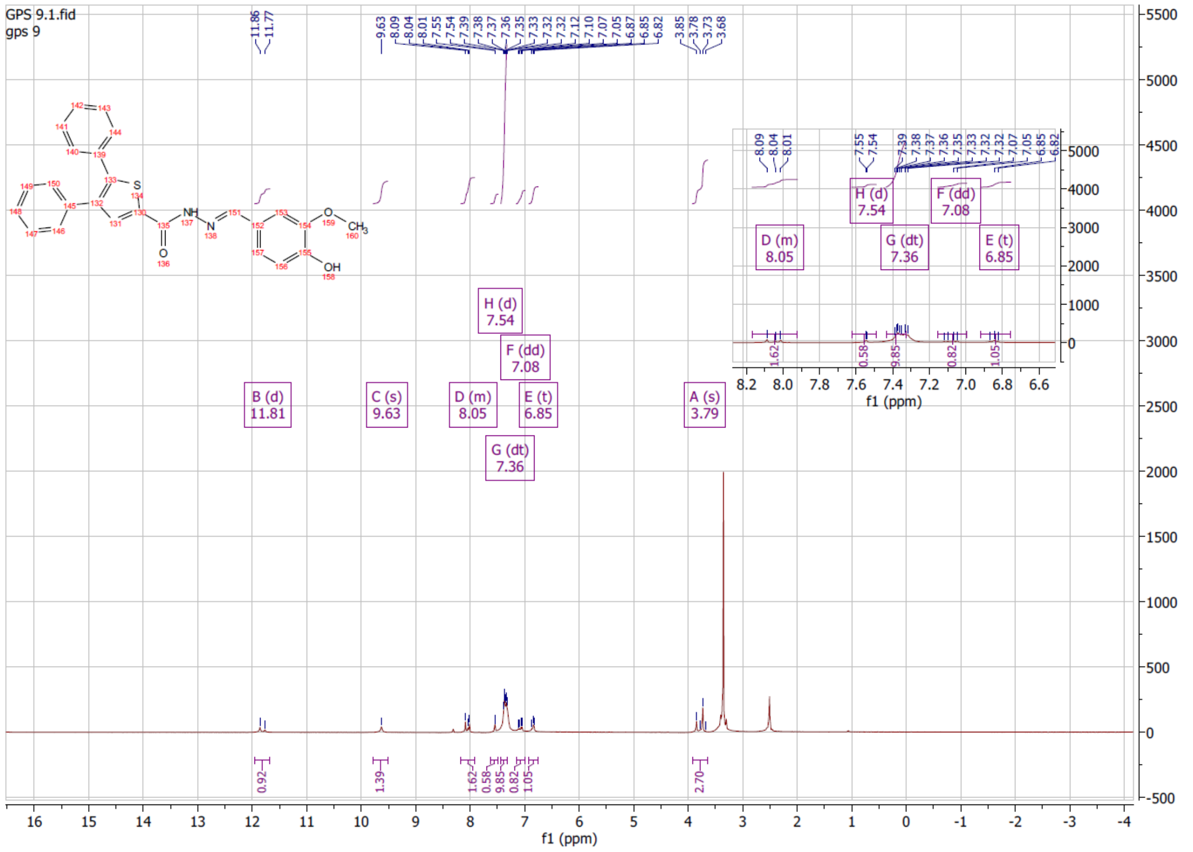

Figure S.9.-^{1}H-NMR spectrum of compound 7e

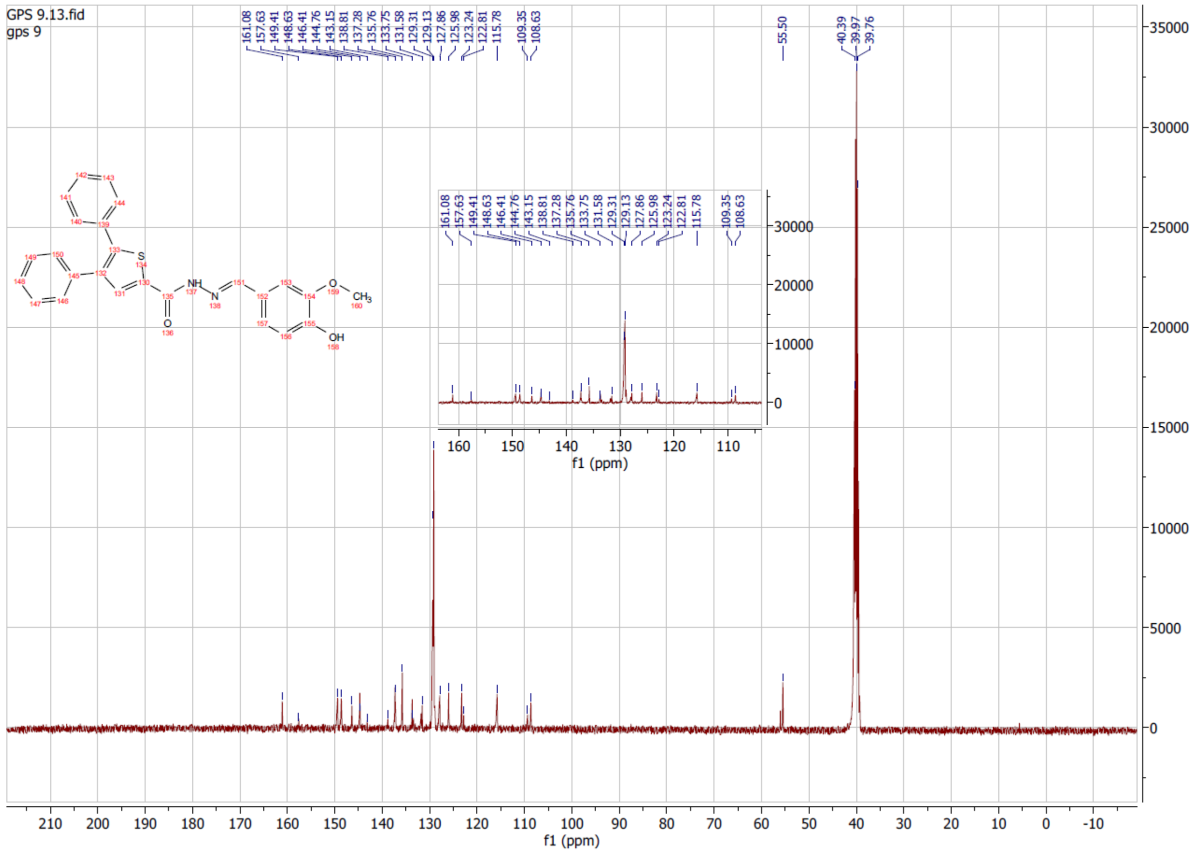

Figure S.10. ^{13}C-NMR spectrum of compound 7e

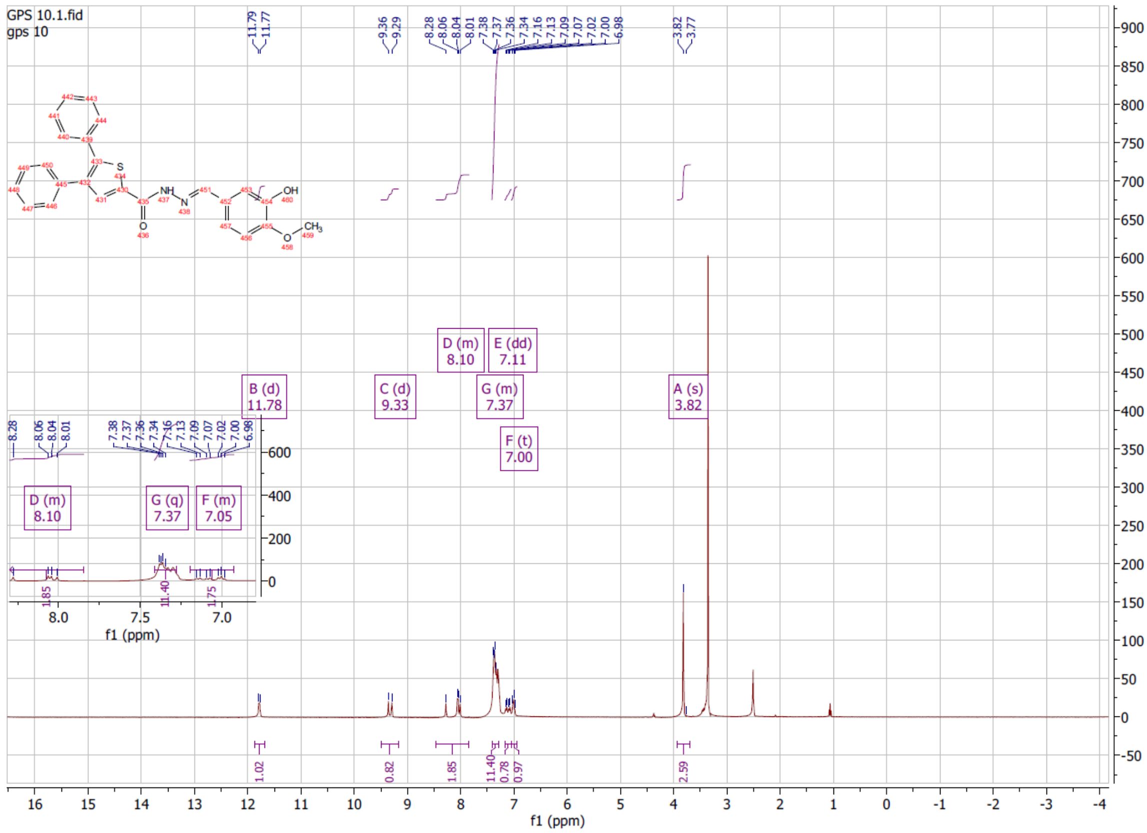

Figure S.11.-^{1}H-NMR spectrum of compound 7f

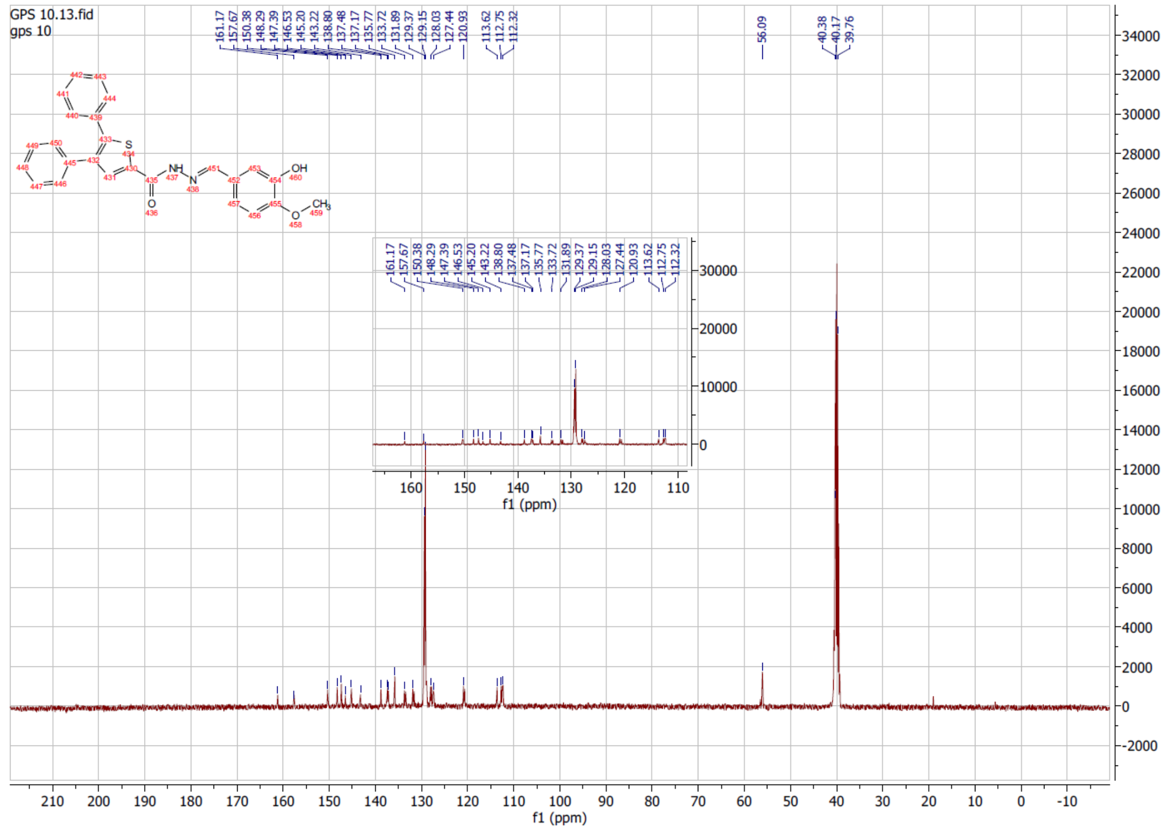

Figure S.12. ^{13}C-NMR spectrum of compound 7f

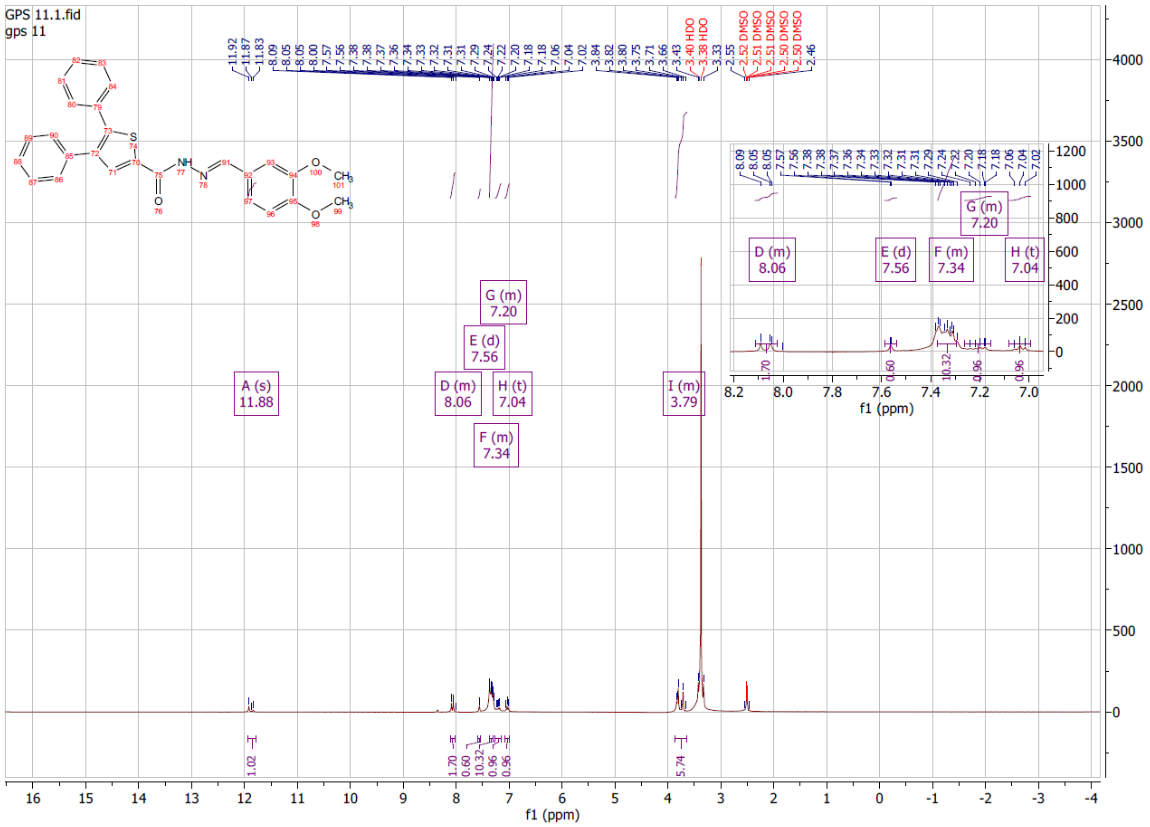

Figure S.13.-^{1}H-NMR spectrum of compound 7g

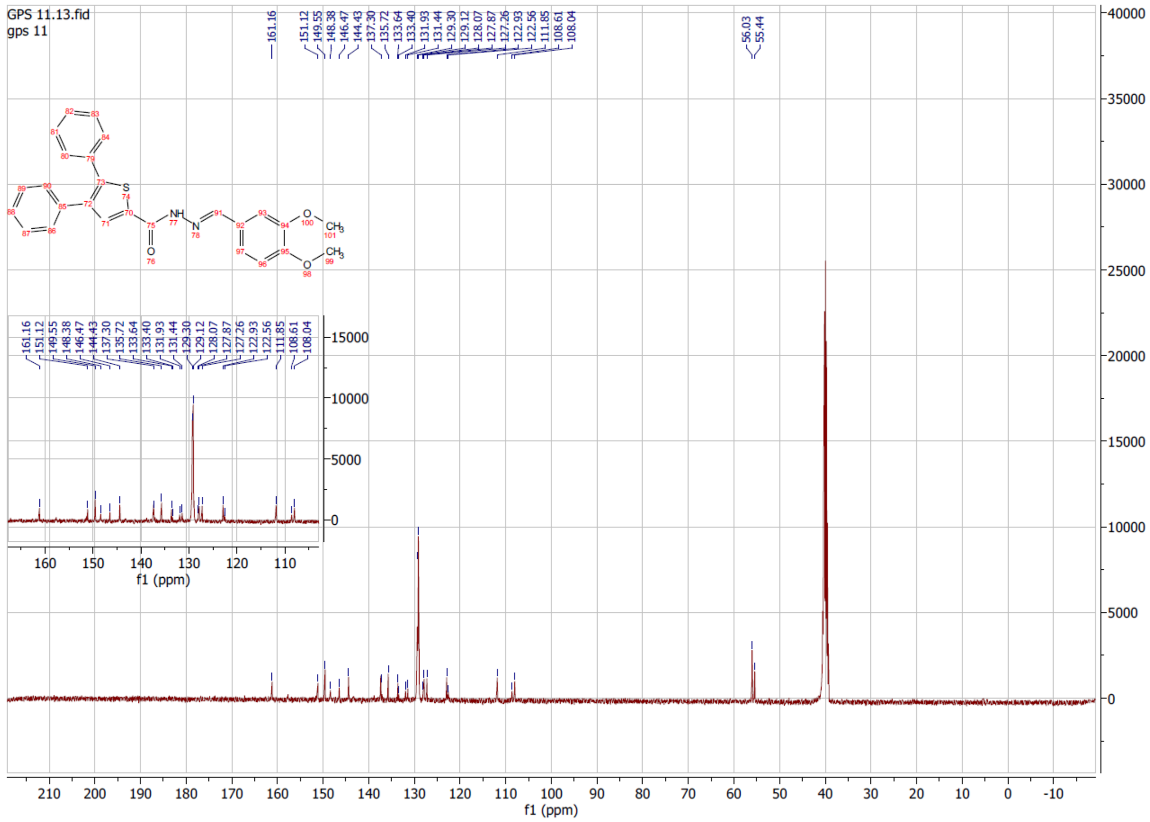

Figure S.14. ^{13}C-NMR spectrum of compound 7g

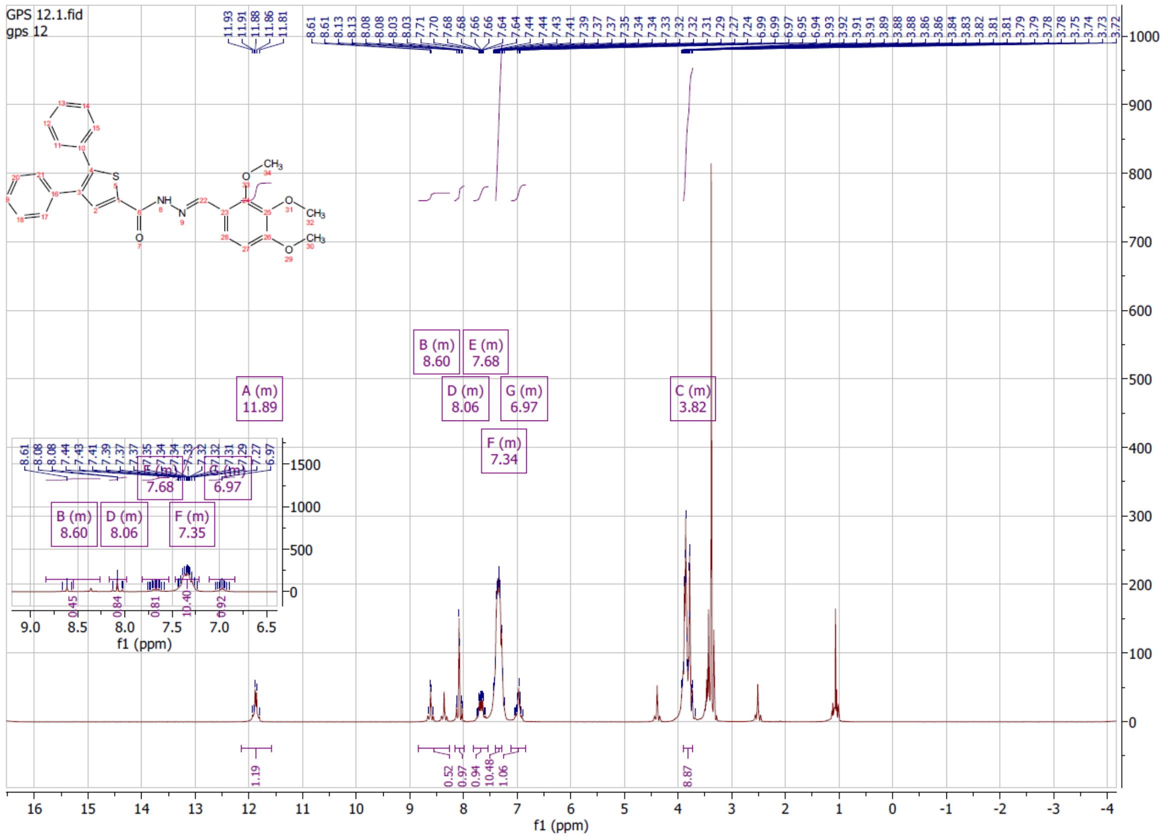

Figure S.15.-^{1}H-NMR spectrum of compound 7h

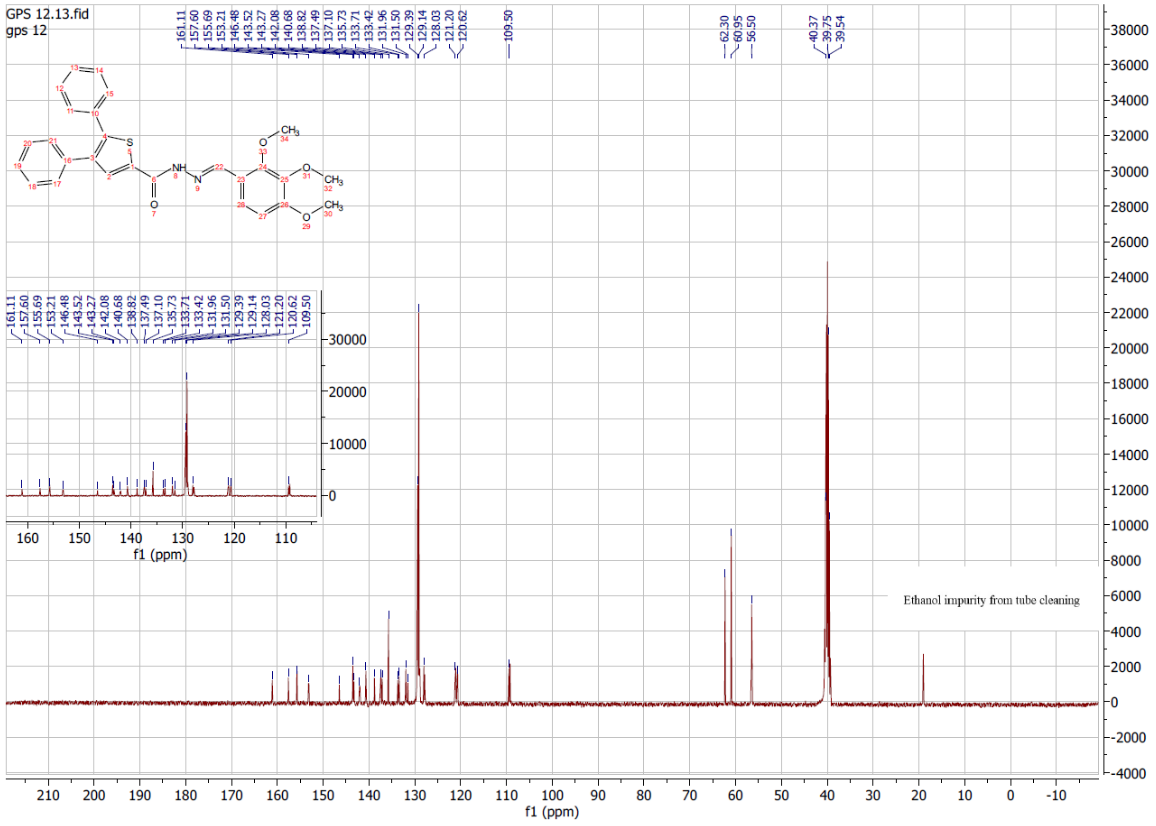

Figure S.16. ^{13}C-NMR spectrum of compound 7h

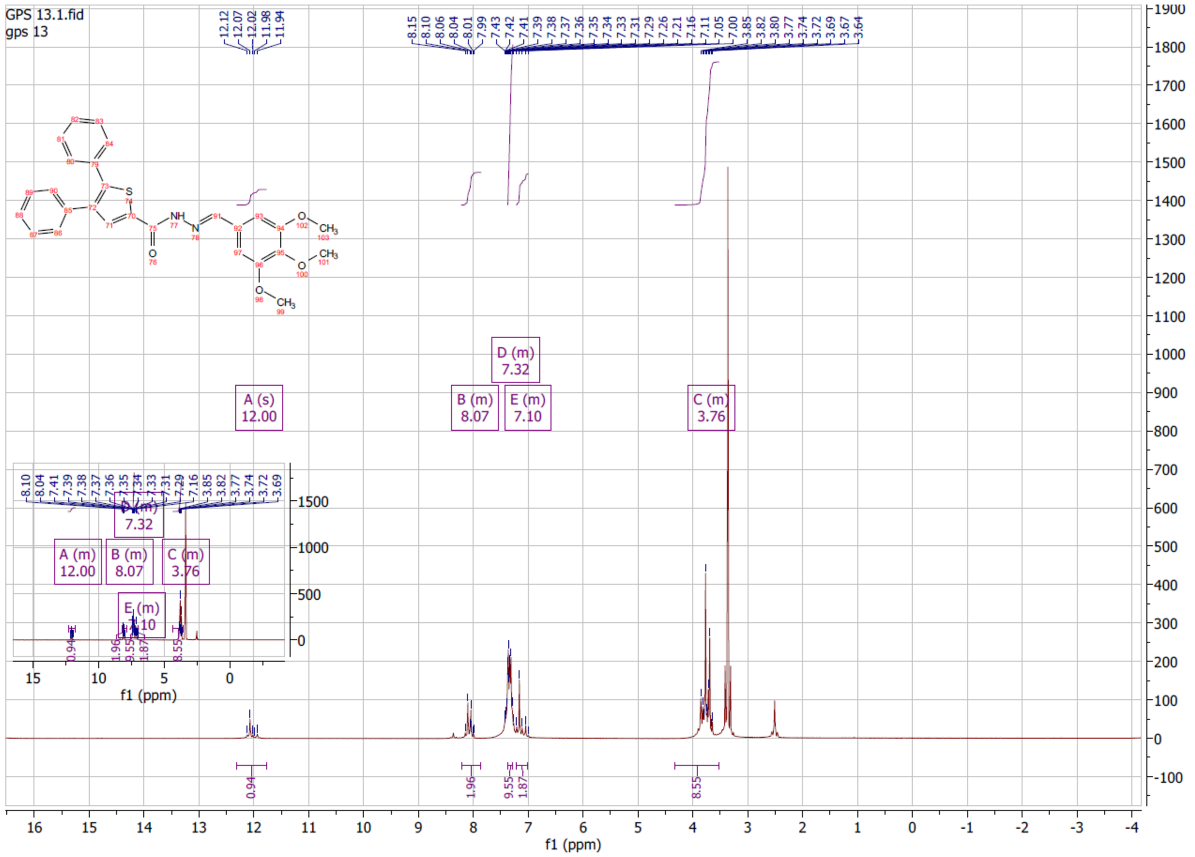

Figure S.17.-^{1}H-NMR spectrum of compound 7i

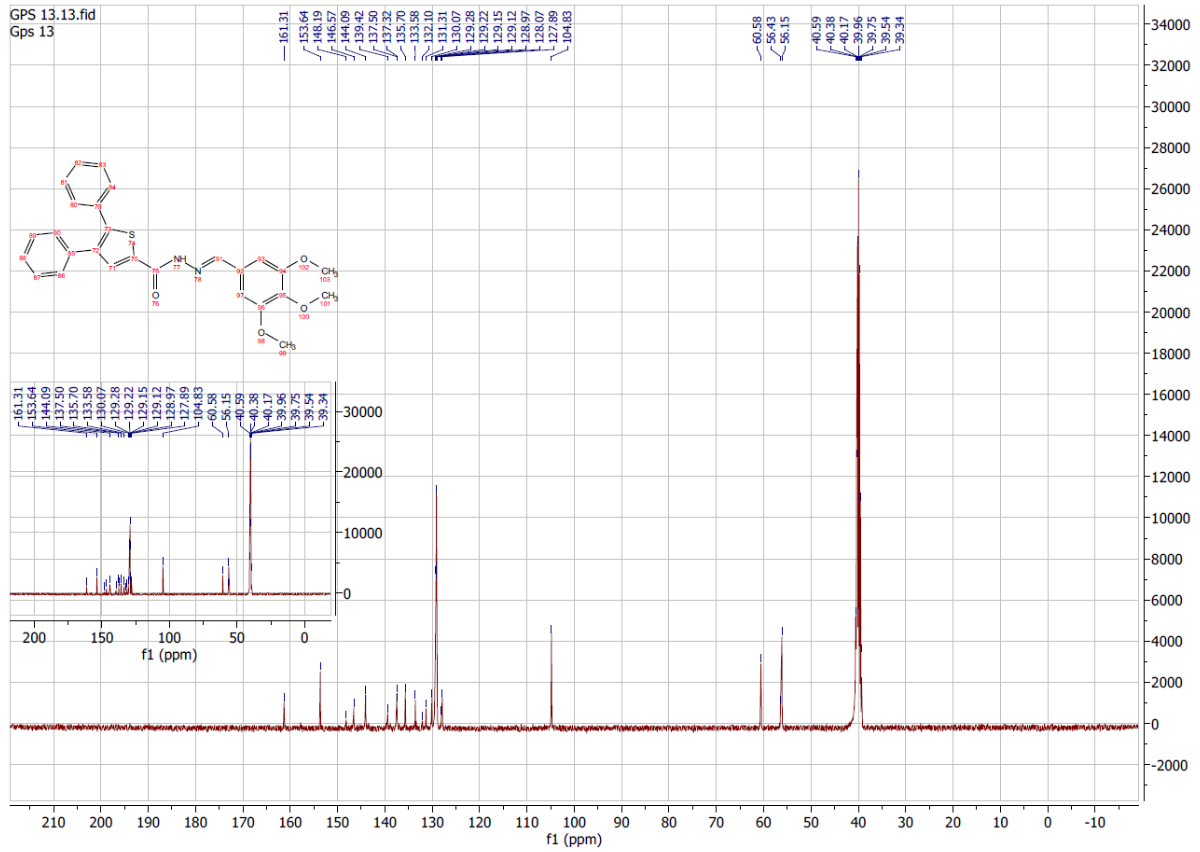

Figure S.18. ^{13}C-NMR spectrum of compound 7i

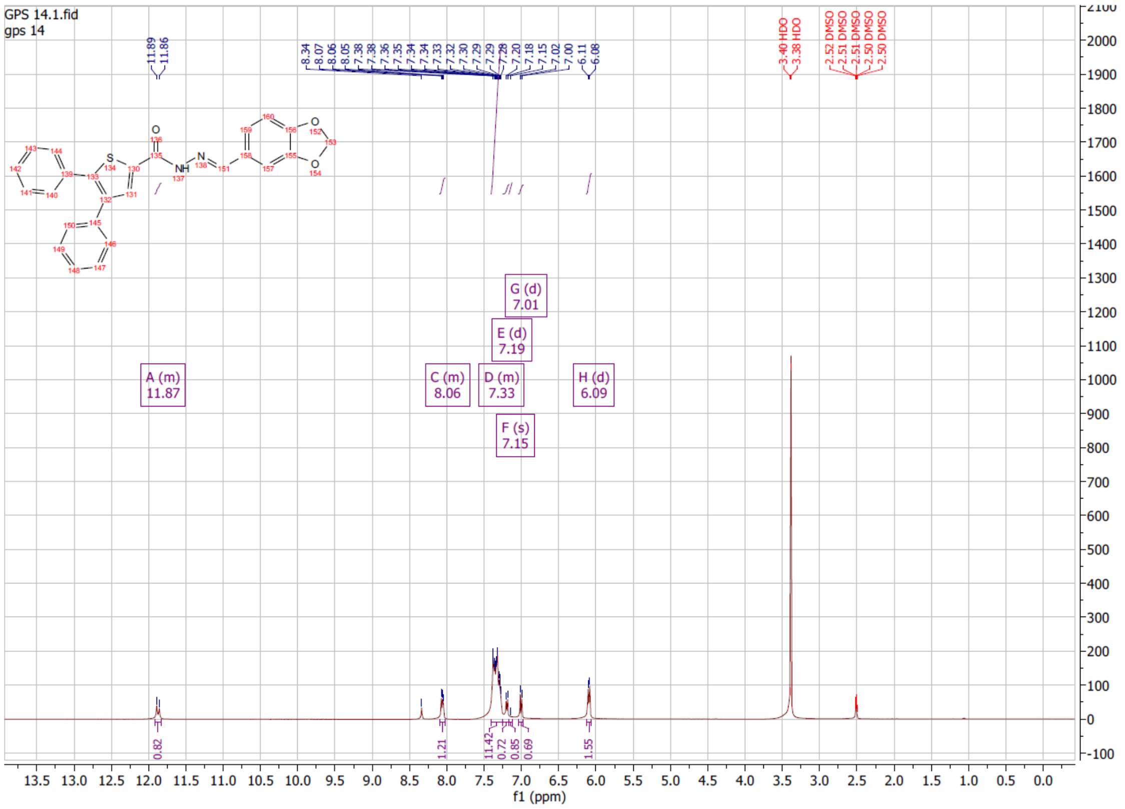

Figure S.19.-^{1}H-NMR spectrum of compound 7j

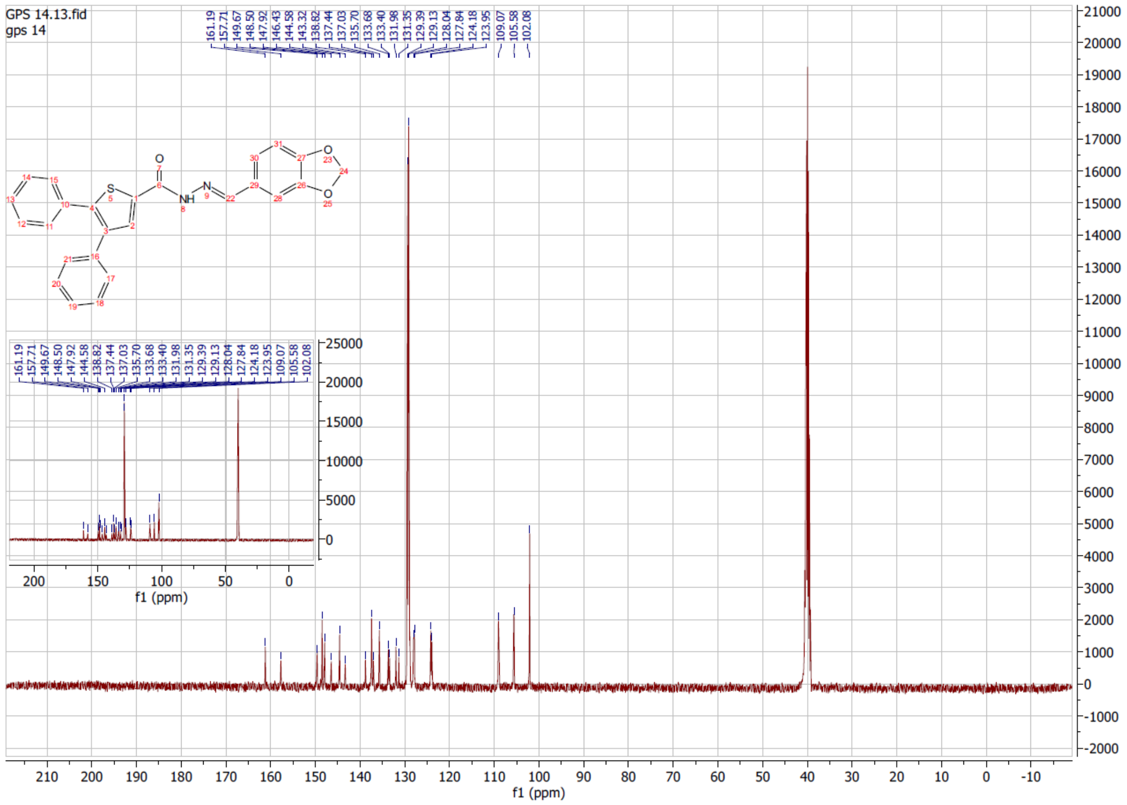

Figure S.20. ^{13}C-NMR spectrum of compound 7j

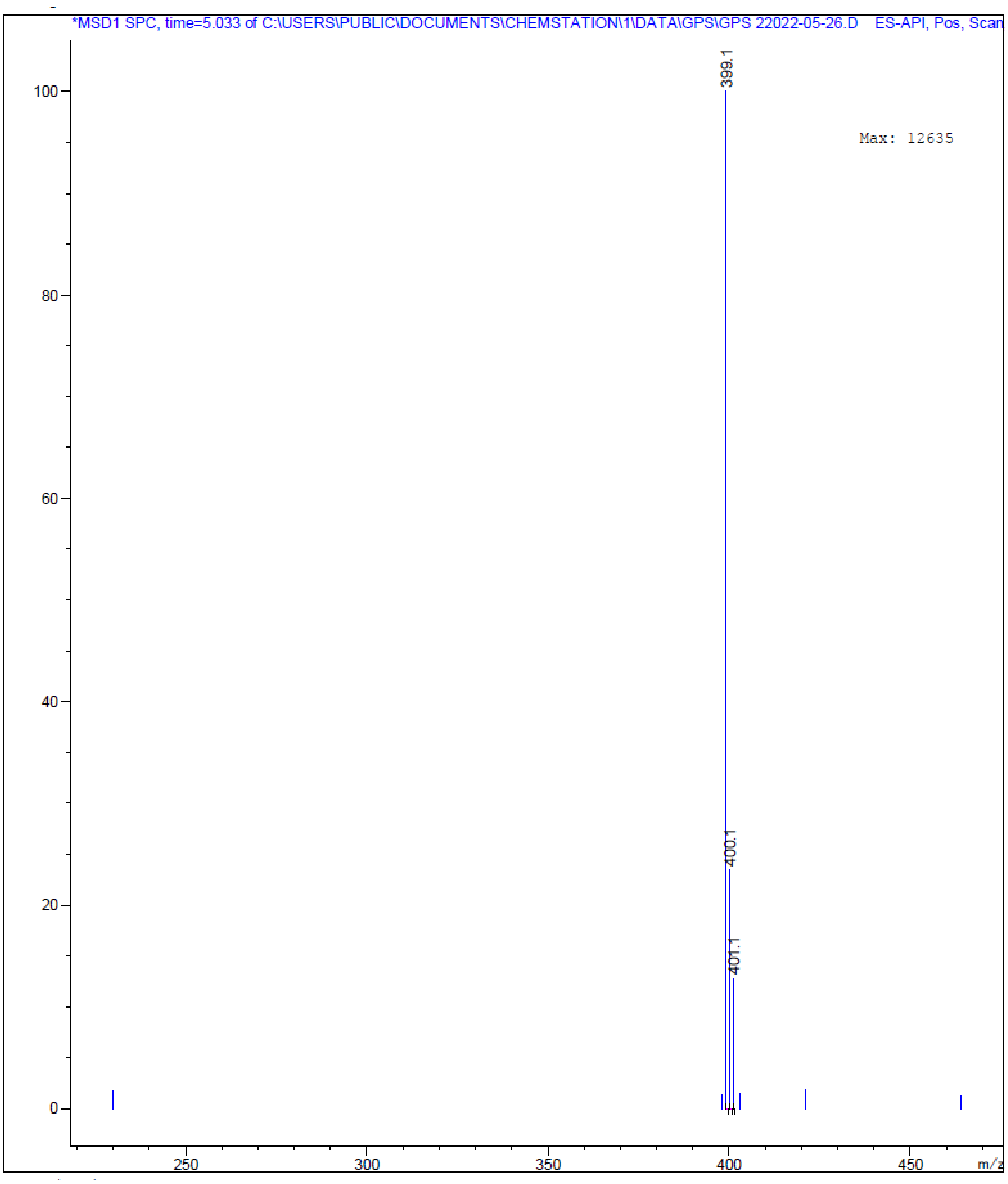

Figure S.21. LC-MS spectrum of compound 7a

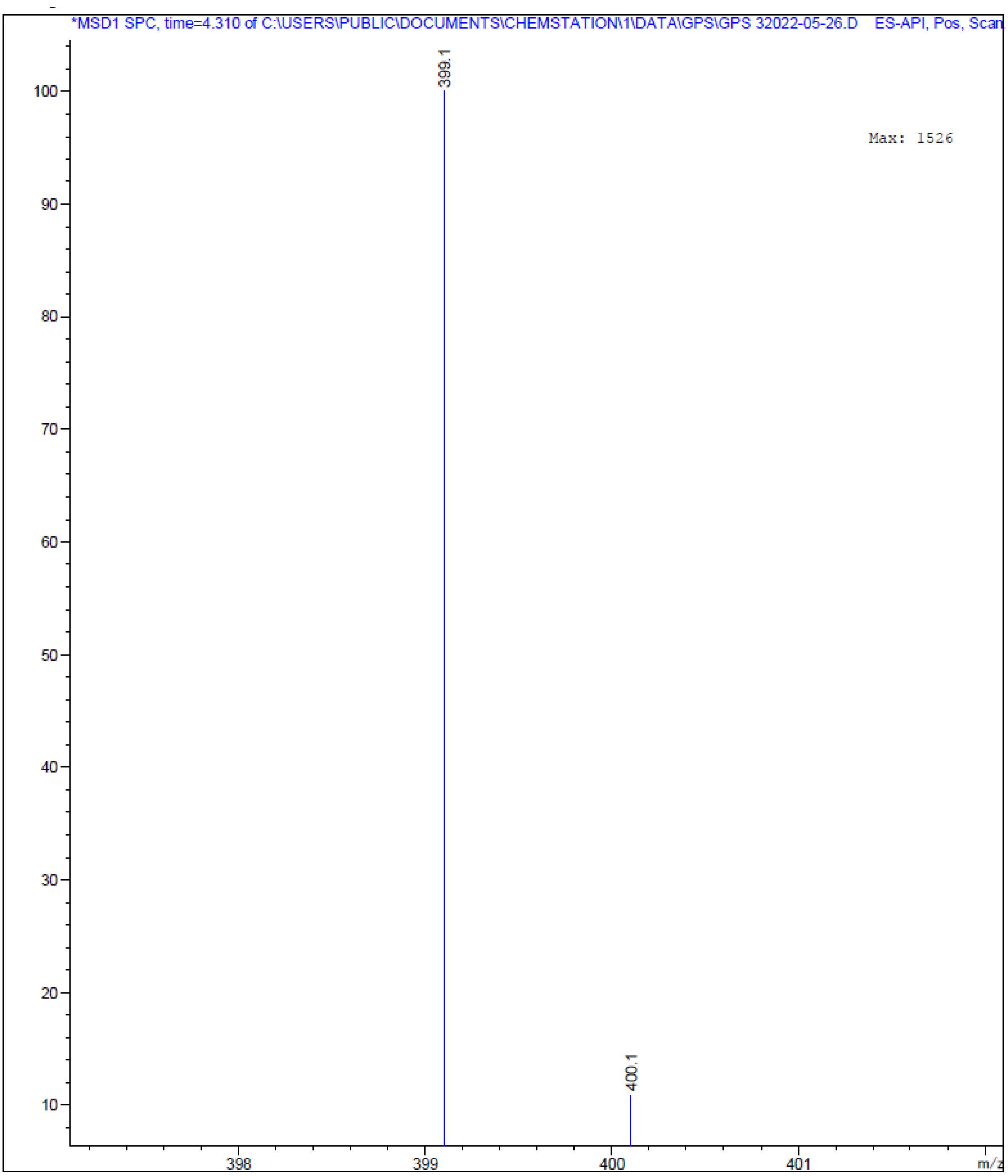

Figure S.22. LC-MS spectrum of compound 7b

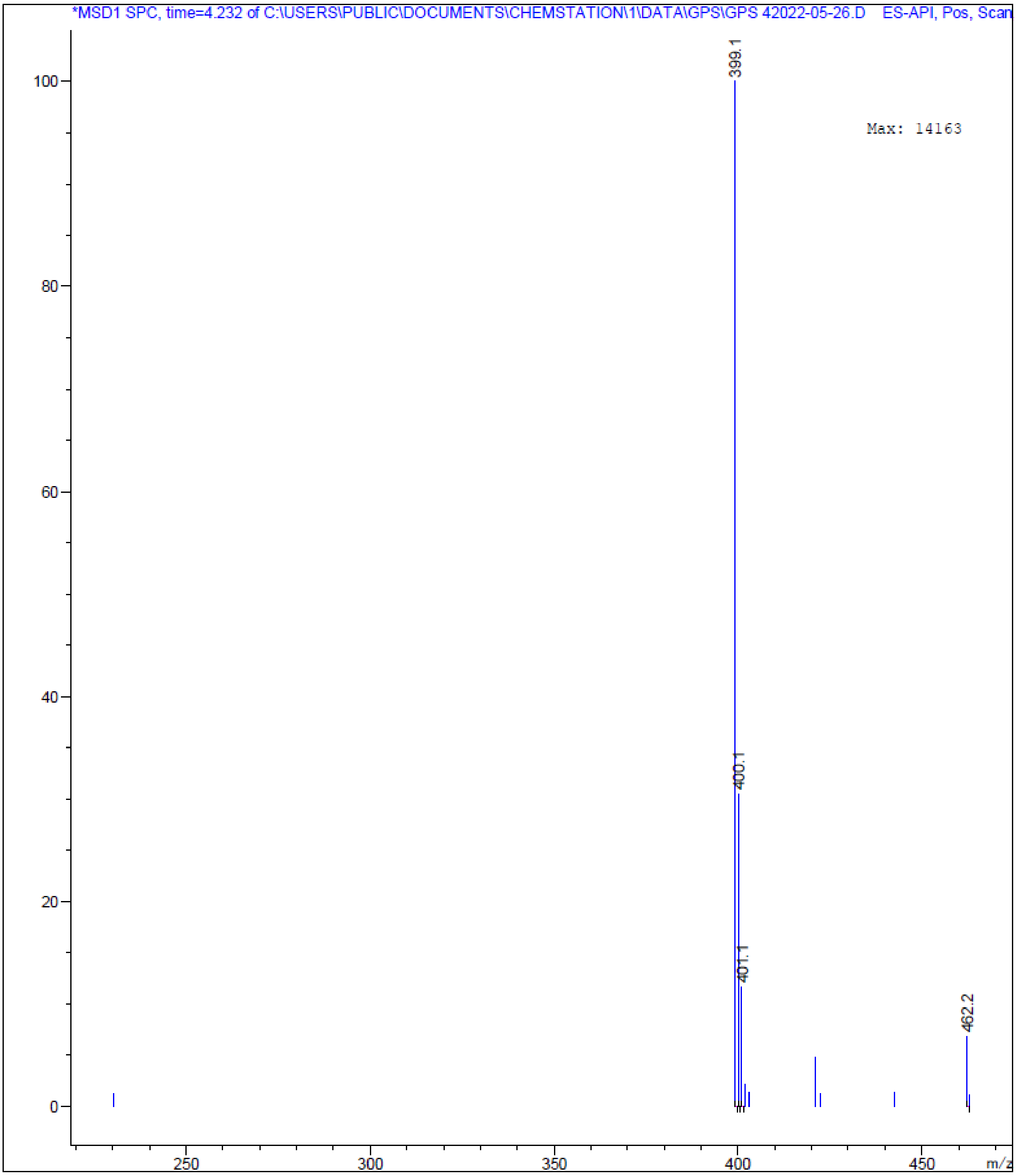

Figure S.23. LC-MS spectrum of compound 7c

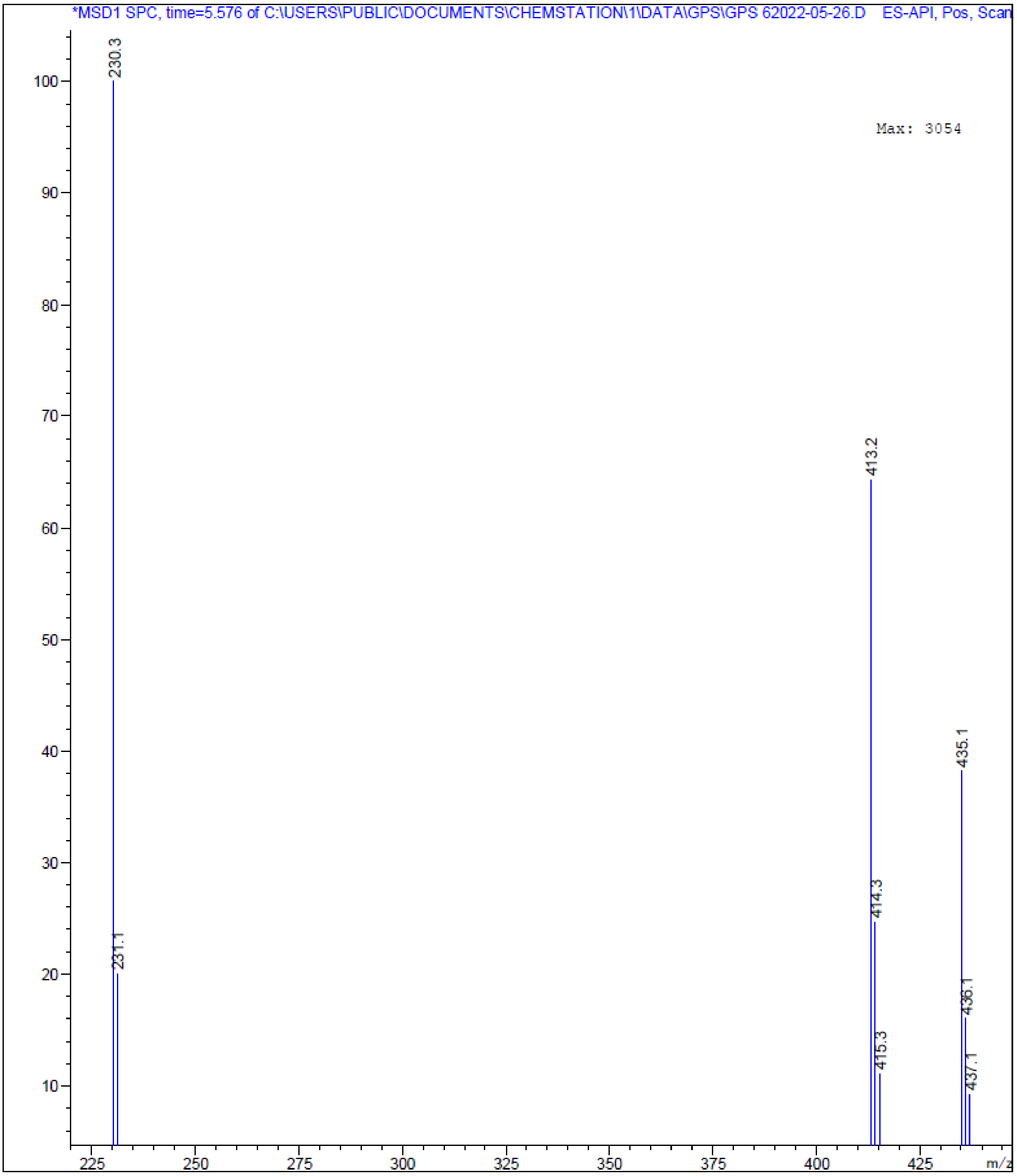

Figure S.24. LC-MS spectrum of compound 7d

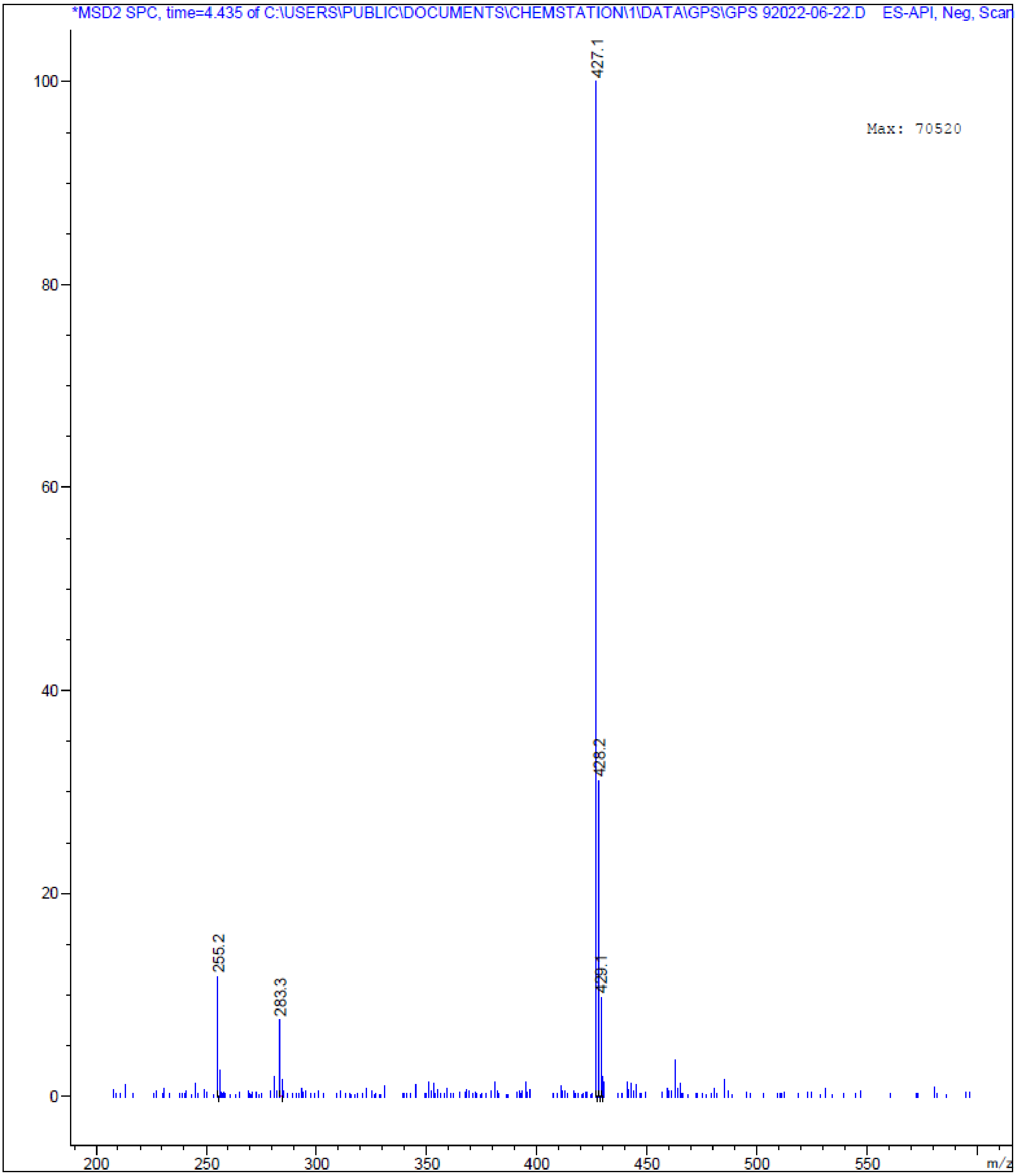

Figure S.25. LC-MS spectrum of compound 7e

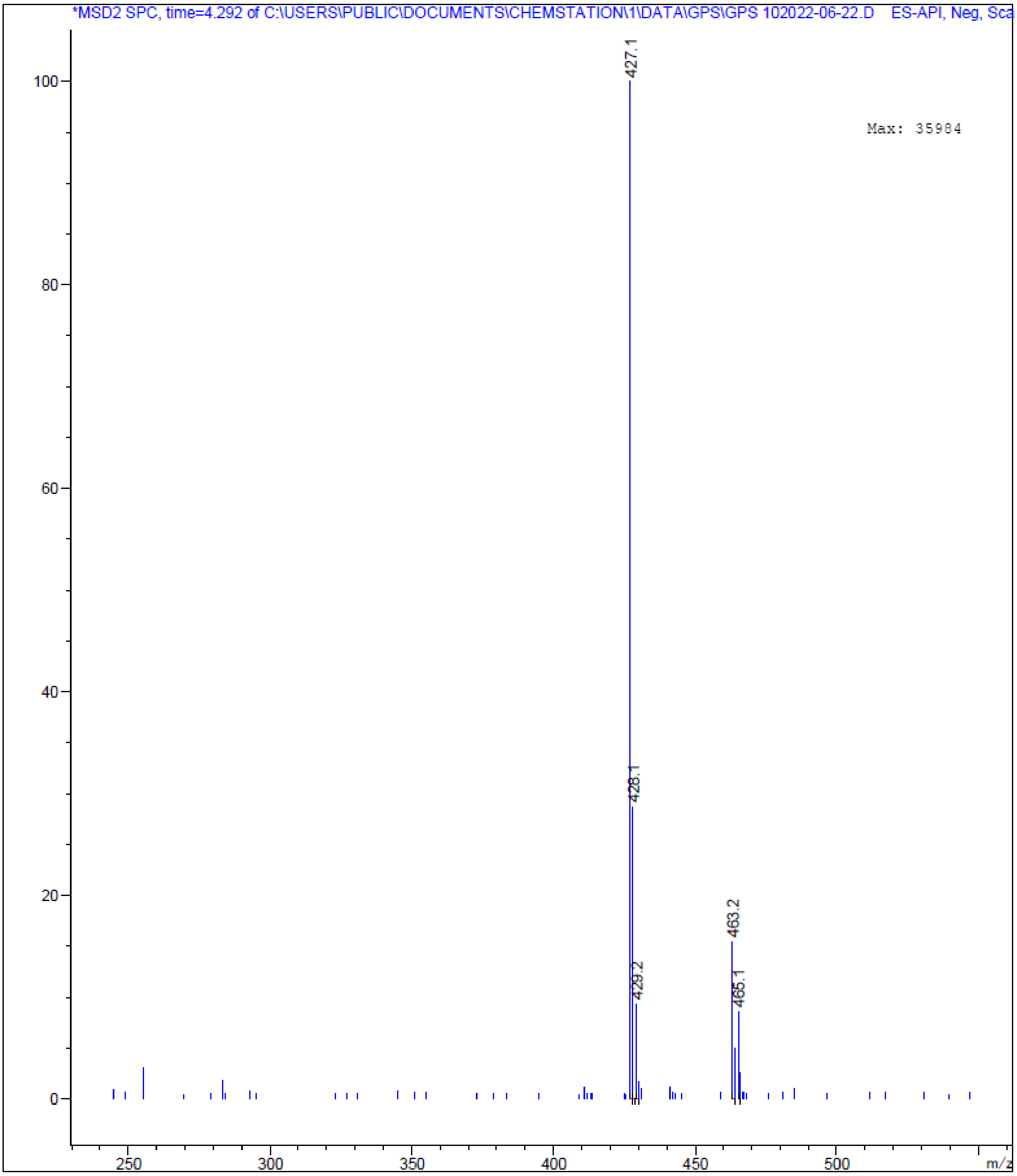

Figure S.26. LC-MS spectrum of compound 7f

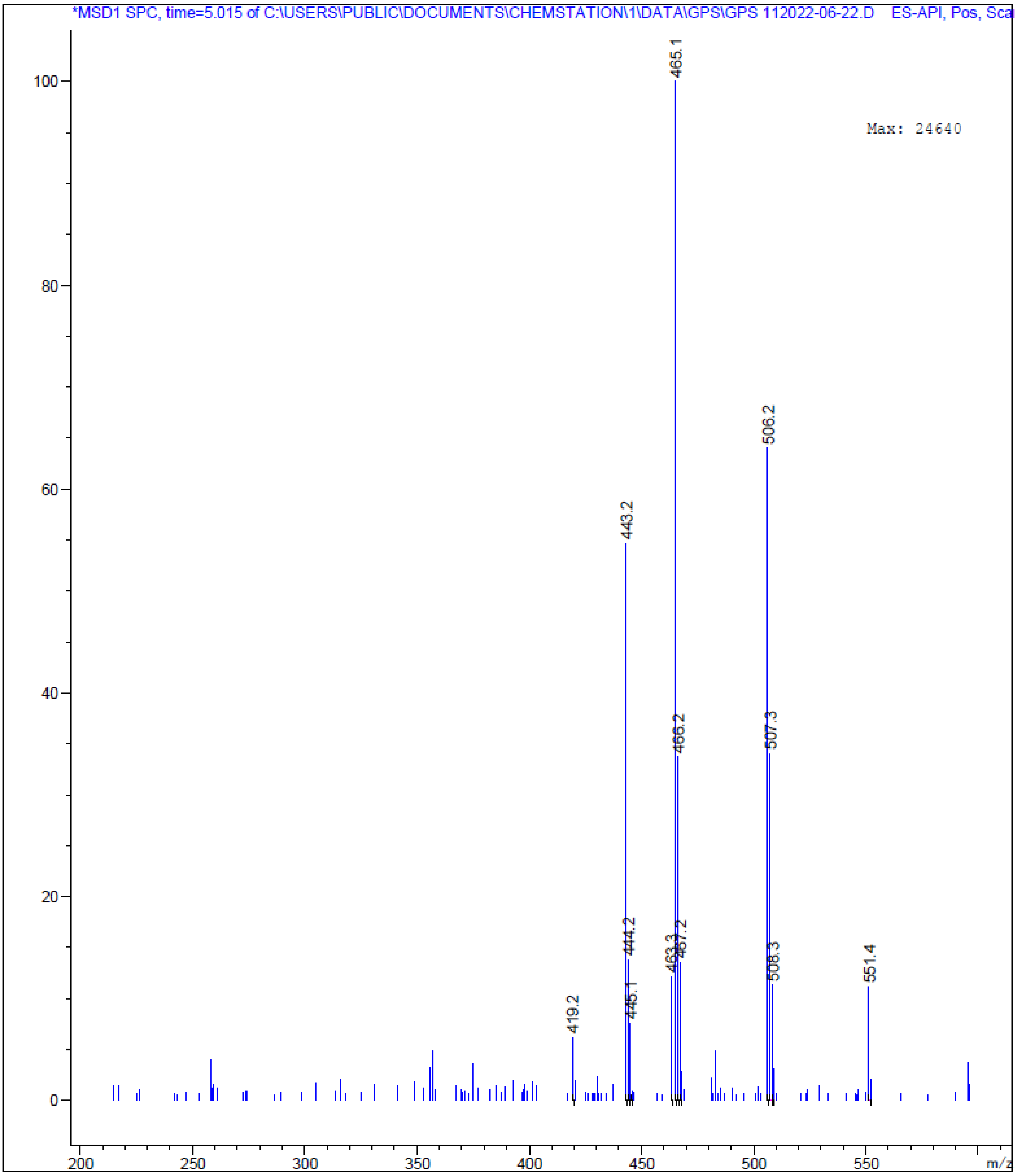

Figure S.27. LC-MS spectrum of compound 7g

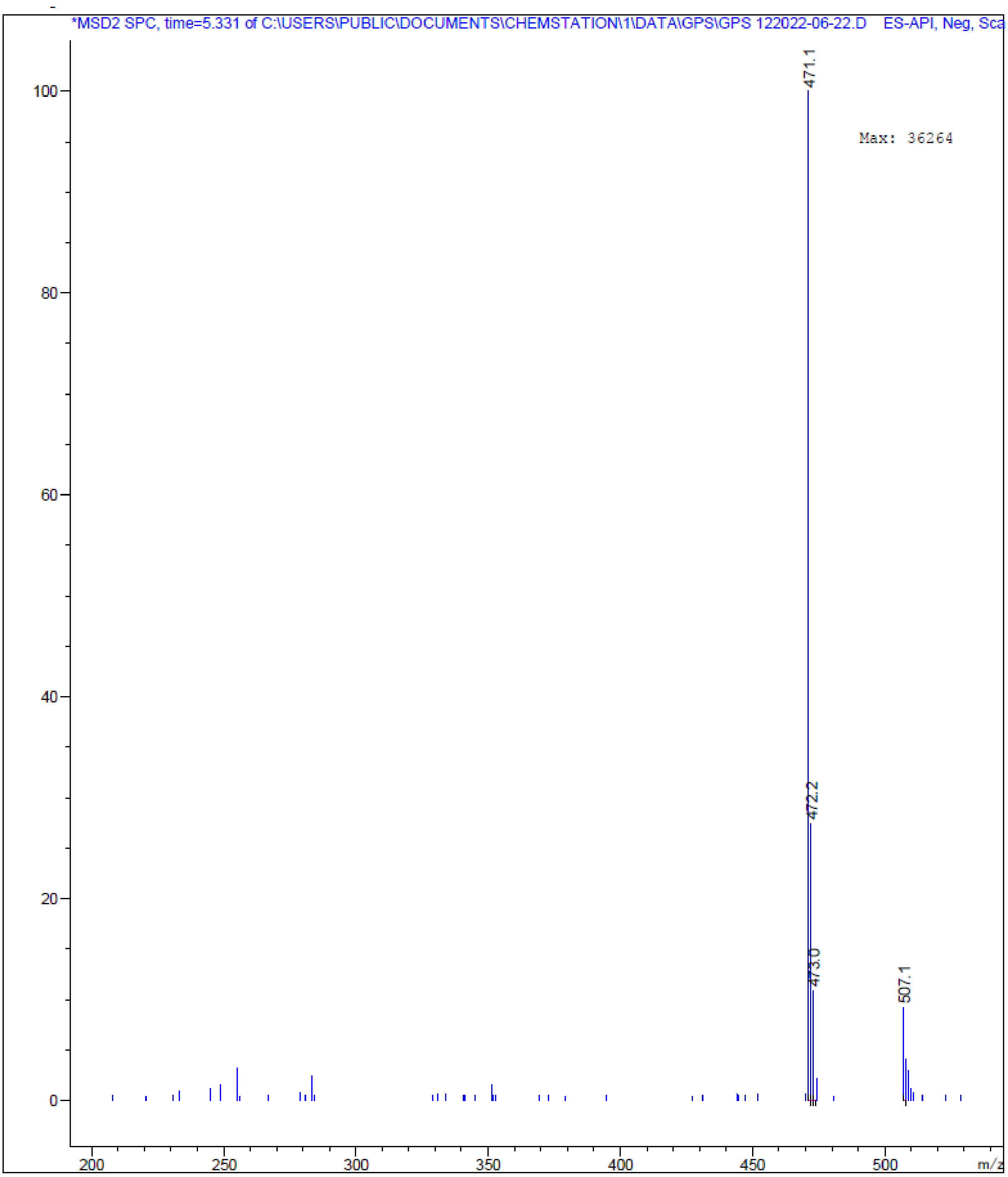

Figure S.28. LC-MS spectrum of compound 7h

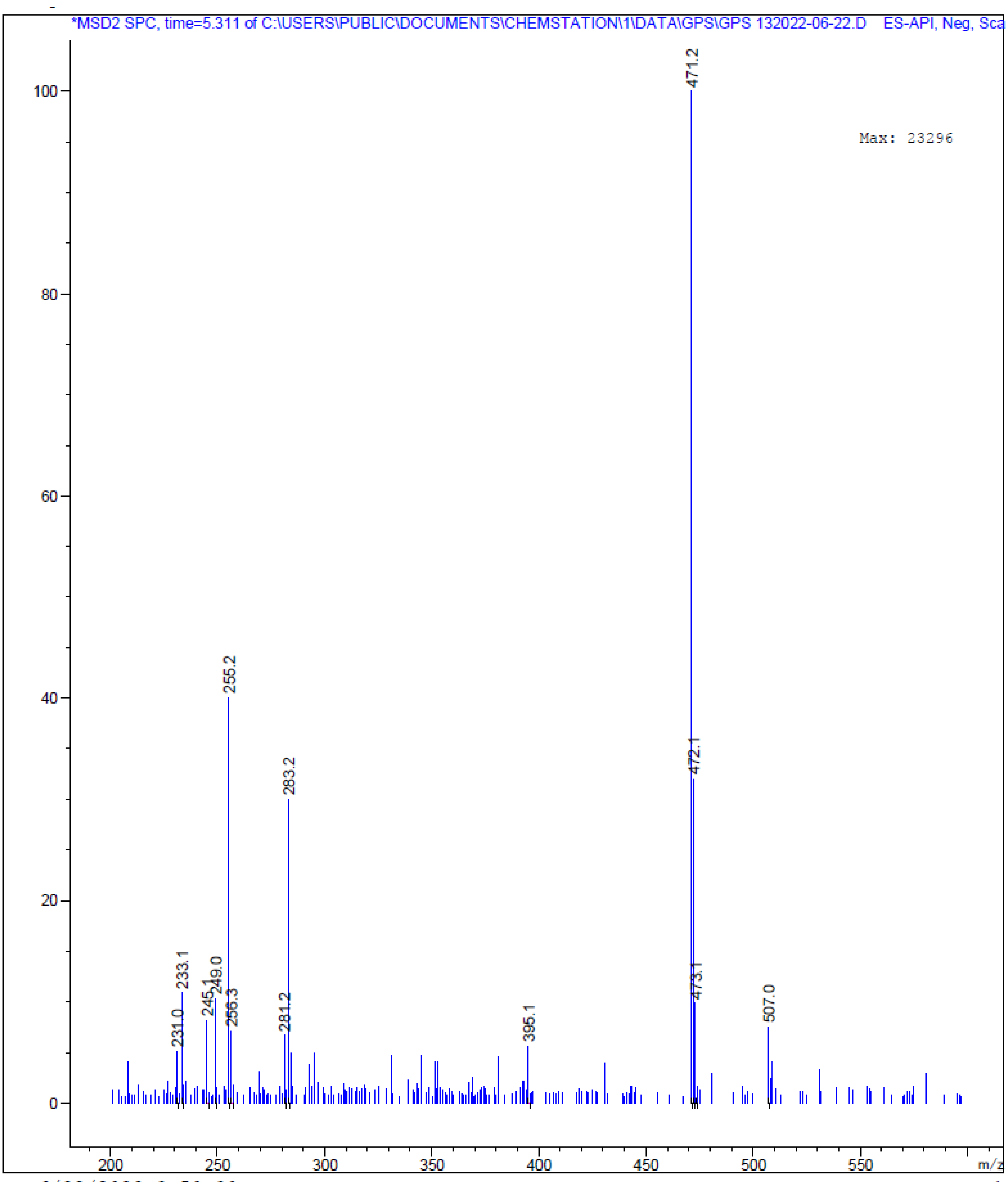

Figure S.29. LC-MS spectrum of compound 7i

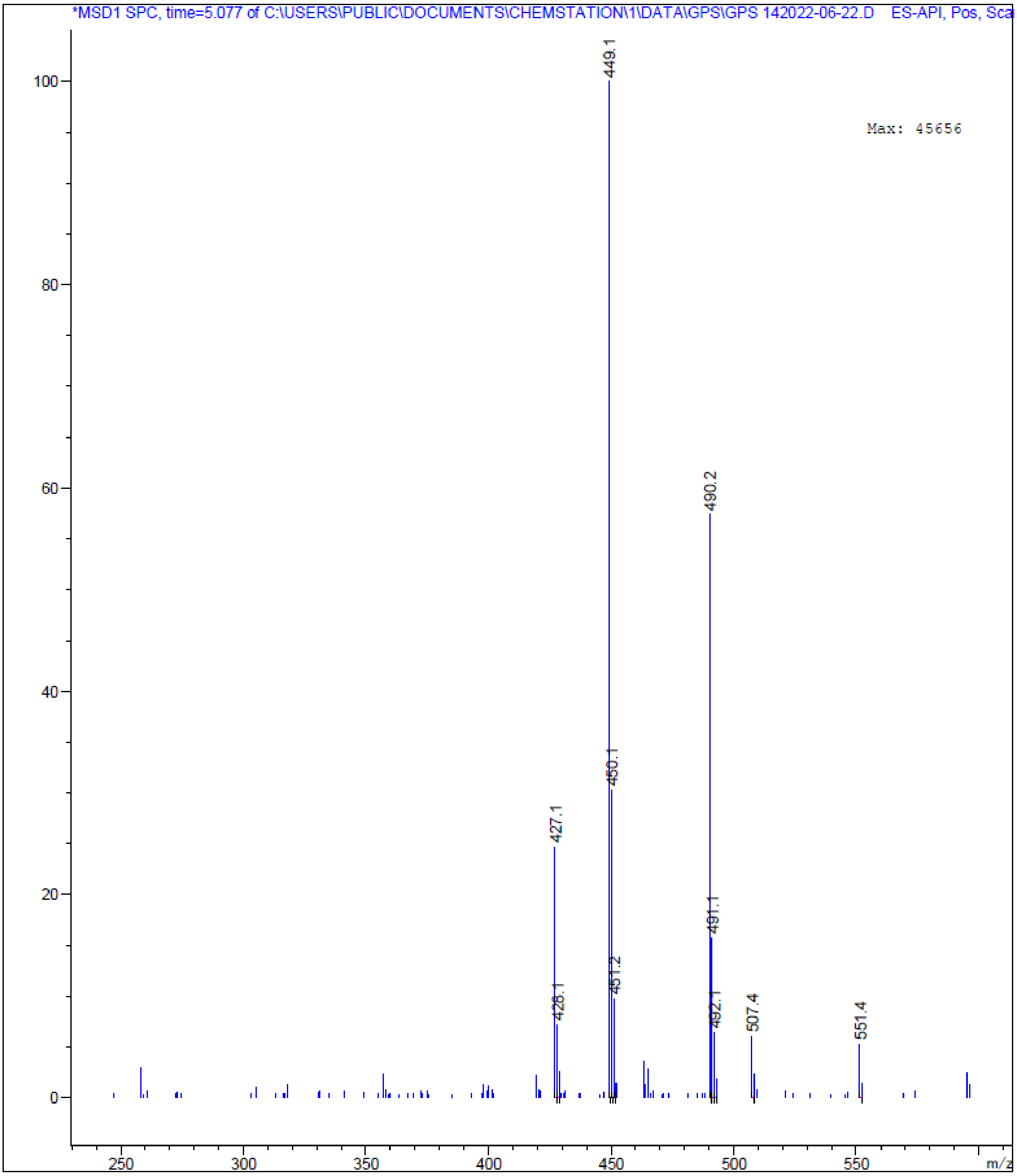

Figure S.30. LC-MS spectrum of compound 7j
